# Successful Liposteroid Therapy for a Recurrent Idiopathic Pulmonary Hemosiderosis with Down Syndrome

**DOI:** 10.1155/2020/5292947

**Published:** 2020-04-25

**Authors:** Hiromi Tobai, Jun Yano, Norio Sato, Fumitaka Amanuma, Mikio Takahashi, Mikiya Endo, Masataka Ishimura, Shouichi Ohga, Hidekazu Maruyama

**Affiliations:** ^1^Department of Pediatrics, Iwate Prefectural Iwai Hospital, Ichinoseki, Japan; ^2^Department of Pediatrics, Graduate School of Medical Sciences, Kyusyu University, Fukuoka, Japan; ^3^Department of Clinical Laboratory, Iwate Prefectural Iwai Hospital, Ichinoseki, Japan; ^4^Department of Pediatrics, Iwate Medical University, School of Medicine, Morioka, Japan

## Abstract

Idiopathic pulmonary hemosiderosis (IPH) is a rare and life-threatening disorder. Early diagnosis and appropriate management are essential for their better prognosis and patients' quality of life (QOL). It is considered that Down syndrome patients with IPH have a worse prognosis compared to other IPH cases. A 2-year-old girl with Down syndrome received the diagnosis of IPH after two episodes of massive pulmonary hemorrhage requiring assist ventilation, who suffered from recurrent IPH during tapering period of oral corticosteroid, started liposteroid therapy. We report here a case of successful control of recurrent IPH and improved QOL enormously with tapering dose of corticosteroid after starting liposteroid therapy.

## 1. Introduction

Idiopathic pulmonary hemosiderosis (IPH) is a rare disorder that is characterized by the triad of hemoptysis, iron deficiency anemia, and diffuse pulmonary infiltrates on chest radiographs. It occurs more frequently in children, usually is diagnosed before at the age of 10 years, although may occur later in life [1, 2]. The etiology of the disease remains unknown and several hypotheses have been reported: autoimmune, allergic, genetic, or environmental hypothesis [1, 3, 4]. The gold standard for IPH diagnosis is lung biopsy, but this method is challenging due to its invasive nature and potential complications, especially in young children [2, 5]. Other diagnostic methods can be conducted for confirmation of hemosiderin-laden macrophages (siderophages) by bronchoalveolar lavage, sputum, or gastric lavage analysis [5–7]. Systemic corticosteroid is the first line treatment of IPH for acute bleeding [2, 6, 8, 9]. Immunosuppressants, including azathioprine, hydroxychloroquine, and cyclophosphamide have been proposed in patients with unfavorable response to corticosteroid [1, 4, 10, 11]. However, long-term use of these drugs is associated with many adverse effects and their use should be to limited to the minimum duration and the dosage necessary [12]. Various therapeutic trials have attempted to improve the prognosis with IPH. Nevertheless, no effective maintenance therapy has been established for children with refractory IPH [7, 8]. Liposteroid, dexamethasone palmitate, has been introduced as a new effective therapy [8].

Through our case report, we discuss the importance of early diagnosis and management of refractory IPH in Down syndrome, who started liposteroid therapy after the recurrent bleeding with tapering of oral prednisolone and successfully controlled the disease.

## 2. Case Report

A 2-year-old girl with Down Syndrome was admitted to our hospital, with weakness, pail, and fever. She presented dyspnea, tachypnea, and severe anemia with hemoglobin level of 2.2 g/dl.

She was born at term, and entered the neonatal intensive care unit (NICU) with mild respiratory distress for five days. She received the diagnosis of Trisomy 21 until the disharge of NICU. There was no cardiac, gastrointestinal, or hematologic disease. Two years after then, prolonged routine follow-up showed good clinical course, although growth and development were below age appropriate milestones.

**Figure 1:**
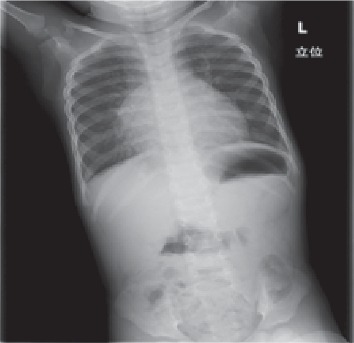
Posteroanterior chest radiograph showing reticular micronodular opacities bilaterally in the first visit.

Upon admission in our ward, physical examination revealed body weight : 7720 g (−2.9 SD), height : 76.3 cm (−2.9 SD), heart rate : 140 bpm, oxygen saturation in room air 90%, and body temperature : 38.0°C. Laboratory examination we observed; severe anemia as mentioned above, red blood cells (RBC): 1.24 × 10^6^/*μ*l, hematocrit value: 7.9%, reticulocyte count: 6%, with normal white blood cells (WBC) and platelet count. The value of mean corpuscular volume (MCV): 63.4 fl, mean corpuscular hemoglobin (MCH): 17.7 pg/cell, mean corpuscular hemoglobin concentration (MCHC): 27.9 g/dl, and serum iron (10 *μ*g/dl) were very low. Plasma ferritin level (15.8 ng/ml) was within the normal range for patient's age. The coagulation tests, renal function, electrolytes, and liver function were unremarkable. Antiglobulin tests were negative and haptoglobin level was normal. Serum immunoglobulin levels were within the normal range, and the serologic tests for autoimmune diseases; antinuclear antibodies (ANA), anti-ds DNA antibodies, anti-cyclic citrullinated peptide (anti-CCP) antibodies, anti-Sm antibodies, and rheumatoid factor (RF) were all negative. Chest X-ray indicated bilateral interstitial reticular infiltrates (Figure 1). Thoracic CT scan was performed and revealed nodular opacities in the right lung. A bone marrow aspiration revealed erythrocyte hyperplasia without malignant cells or hemophagocytic cells. Although no definite diagnosis was obtained, packed red blood cell transfusion was administered, and oral iron was started.

**Figure 2:**
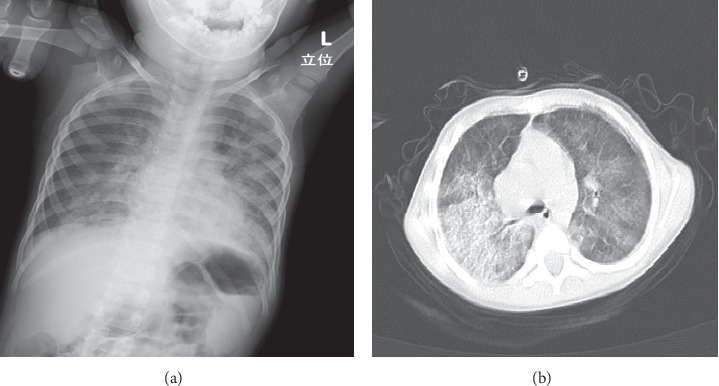
(a) Posteroanterior chest radiography demonstrating bilateral pulmonary infiltrations at three months after first acute alveolar hemorrhage. (b) Axial CT scan of the thorax showing diffuse ground-glass appearance.

**Figure 3:**
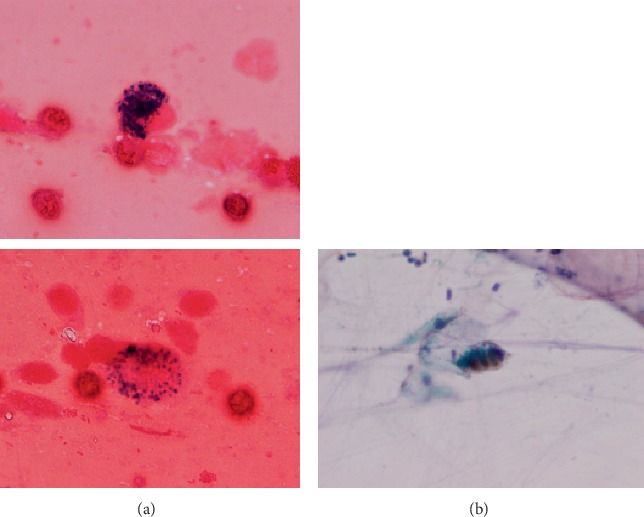
(a) Hemosiderin-laden macrophage within gastric lavage fluid (hematoxylin and eosin stain). (b) Prussian blue stain of gastric lavage fluid.

Three months later, she was again admitted to the hospital for severe anemia with bilateral alveolar infiltrates on chest X-ray (Figure 2(a)). Repeated thoracic CT showed widespread ground-glass appearance throughout both lungs (Figure 2(b)). She presented cough, tachypnea, pulse oximetry indicating 60% saturation, and wheezing with auscultation of respiratory failure. Subsequently, she was transferred to pediatric intensive care unit (PICU) and required tracheal intubation and mechanical ventilation. Then, the same episode as above occurred 4 months later. At that time, IPH was suspected from clinical manifestation and radiologic findings, and a diagnosis of IPH was confirmed by gastric lavage fluid that demonstrated the presence of hemosiderin-laden macrophages (Figures 3(a) and 3(b)). Intravenous prednisolone (2 mg/kg/day) and blood transfusion were given immediately for acute pulmonary bleeding, and promptly improved clinical symptoms and laboratory data. Then, oral prednisolone was started as maintenance therapy. However, weaning the dose of prednisolone failed to control the disease, and the episodic pulmonary hemorrhages developed every 3-4 months. For this reason, increasing the dose was resumed. She progressively became cushingoid due to prolonged moderate-to high-dose corticosteroid therapy.

After two and a half years from the onset of IPH, liposteroid was introduced in an attempt to reduce the dose of prednisolone. Liposteroid was intravenously infused at 0.06 mg/kg/day for 3 consecutive days with prednisolone for acute bleeding therapy. After the first liposteroid therapy, the single infusion of the same dose was followed by weekly with tapering prednisolone. She obtained remission three months after initiation of liposteroid even tapering dose of prednisolone, so the interval of infusion was changed step by step, and she is now on liposteroid every 4 weeks and low dose of prednisolone 0.18 mg/kg/day with better controlled for 21 months. During the following 24 months, she suffered from minor alveolar hemorrhage twice, being triggered by respiratory infections, however, her clinical condition improved promptly with 3 consecutive days of liposteroid infusion without increased dose of prednisolone.

## 3. Discussion

IPH is a rare disease with the incidence of 0.24–1.23 cases per million in selected population [2, 13–15]. IPH is life threatening condition and the early diagnosis is essential for early treatment in order to improve the prognosis and to avoid complications of recurrent alveolar hemorrhage. However, its diagnosis may be difficult and is usually delayed due to absence of classical triad (hemoptysis, iron deficiency anemia and diffuse infiltrates on chest X-ray), insidious onset, lack of awareness about the disorder, and the variable clinical courses [2, 10]. There is a long delay (4 months–10 years) between onset of the symptoms and diagnosis [2, 5, 6, 10]. Especially, in young children, hemoptysis is not common as the first symptom of IPH, occurred in about 50% of patients [10, 16], who swallow hemorrhage sputum. In many reports, shortness of breath, iron deficiency anemia, and alveolar infiltration on chest X-ray are typically seen [17]. Iron deficiency anemia may be the first and only manifestation with the lack of hemoptysis. Also, pulmonary involvement may not be found at the onset of IPH, and chest X-ray may show normally [2]. Plasma ferritin level could be elevated or be in normal limits because of the alveolar synthesis and release into the circulation, and do not reflect the iron deposits of the body [2, 14]. In our case, severe anemia preceded typical clinical symptoms and radiologic findings of IPH impeding the ability to make a more rapid diagnosis. Greater awareness such as clinical suspicion for the diagnosis of IPH in patients with repeated iron deficiency anemia may lead to the earlier diagnosis and appropriate management, thereby lessening or entirely avoiding major complications.

Corticosteroids are suggested as the first line treatment for acute episodes of alveolar bleeding. In many case reports, corticosteroids were initiated with rapid improvement in clinical course [4, 6, 18]. On the other hand, their effect on the chronic phase is unclear and their effects on the prognosis remain still controversial. Furthermore, prolonged corticosteroid therapy results in cushingoid feature, weight gain, osteoporosis, and growth retardation [9, 11]. Immunosuppressive agents are the second choice of drugs, especially in steroid-dependence or steroid-resistance cases [5, 7, 10, 15, 19]. However, immunosuppressants cause the change of immune system, increase risk of infection that may trigger IPH and the possible risk of developing malignancy [8, 12, 20]. As an another option of treatment, Ohga et al. proposed the liposteroid therapy for the improved outcome of patients with refractory IPH [8, 21].

In our case, corticosteroids therapy was effective in acute bleeding, but recurred bleeding even with the corticosteroid prophylaxis. Moreover, she had a Down syndrome. A French study reported Down syndrome patients with IPH had a worst prognosis compared to others, including a fatal outcome and frequent relapse. They explained that the higher frequency of lower respiratory tract infections in Down syndrome patients is the possible reason of the worst prognosis [10]. It is also well known that an individual with Down syndrome is susceptible to infections, autoimmune disorder and particular types of cancers due to their specific immunity and anatomical reasons [22, 23]. We took into account her poor prognostic factor and immune system, and decided to introduce liposteroid to control her disease.

Dexamethasone has higher affinity for the corticosteroid receptor than prednisolone or methylprednisolone and has stronger anti-inflammatory effects [8]. Liposteroid is dexamethasone palmitate, which is a lipid emulsion containing dexamethasone. Liposteroid has the same mechanism of action as dexamethasone, however, greater efficacy and lower frequency of systemic adverse effects than dexamethasone. In addition, liposteroid has lipo-based, palmitate, which is more harmful to non-adipose tissue, is easily taken up by macrophages, and induces strongly apoptosis of macrophages [24, 25]. Doi et al. suggested that low-dose liposteroid therapy accumulates effectively in the hemorrhagic inflamed sites of the lung, reduces the chance of high-dose corticosteroid therapy and prevents adverse effects [8]. Liposteroid was an agent to prevent acute and chronic bleeding and also contributed to wean steroids in our case. However, our patient has never used immunosuppressant agents, so unable to compare the effect between liposteroid and immunosuppressant agents.

## 4. Conclusion

In conclusion, we emphasize IPH should be suspected by physicians, especially about the diagnosis and management in pediatric patients. Although IPH is a life-threatening disease and causes serious complications, appropriate management is capable of altering the prognosis and patients' quality of life in a positive manner. Our case showed the appropriate diagnosis and management changed patient's clinical course for the better largely, even though she had a poor prognosis of Down syndrome. Liposteroid may be considered as an effective and promising agent for refractory IPH that may limit a patient's cumulative long term exposure to steroids and their resulting complications.
